# Author Correction: Titration of RAS alters senescent state and influences tumour initiation

**DOI:** 10.1038/s41586-026-10601-9

**Published:** 2026-05-01

**Authors:** Adelyne S. L. Chan, Haoran Zhu, Masako Narita, Liam D. Cassidy, Andrew R. J. Young, Camino Bermejo-Rodriguez, Aleksandra T. Janowska, Hung-Chang Chen, Sarah Gough, Naoki Oshimori, Lars Zender, Sarah J. Aitken, Matthew Hoare, Masashi Narita

**Affiliations:** 1 Cancer Research UK Cambridge Institute, Li Ka Shing Centre, University of Cambridge, Cambridge, UK; 2 Department of Molecular and Clinical Cancer Medicine, University of Liverpool, Liverpool, UK; 3 Department of Cell, Developmental and Cancer Biology, Knight Cancer Institute, Oregon Health and Science University, Portland, OR USA; 4 Department of Medical Oncology and Pneumology, University Hospital Tuebingen, Tuebingen, Germany; 5 German Cancer Research Consortium (DKTK), Partner Site Tübingen, German Cancer Research Center (DKFZ), Heidelberg, Germany; 6 iFIT Cluster of Excellence EXC 2180 Image Guided and Functionally Instructed Tumor Therapies, University of Tuebingen, Tuebingen, Germany; 7 Tuebingen Center for Academic Drug Discovery and Development (TüCAD2), Tübingen, Germany; 8 Medical Research Council Toxicology Unit, University of Cambridge, Cambridge, UK; 9 Department of Histopathology, Cambridge University Hospitals NHS Foundation Trust, Cambridge, UK; 10 Early Cancer Institute, Hutchison Research Centre, University of Cambridge, Cambridge, UK; 11 Department of Medicine, University of Cambridge, Cambridge, UK; 12 Tokyo Tech World Research Hub Initiative (WRHI), Institute of Innovative Research, Tokyo Institute of Technology, Yokohama, Japan

**Keywords:** Senescence, Tumour heterogeneity

Correction to: *Nature* 10.1038/s41586-024-07797-z Published online 7 August 2024

In the originally published version of this article, the t-SNE plot coloured by gene expression, labelled “*Cdkn1a*” (p21) in Extended Data Fig. 2e, was incorrect and instead showed “*Cdkn1b*” (p27). This has now been corrected in the HTML and PDF versions of the article to display the appropriate *Cdkn1a* data. This error does not affect the text or conclusions of the study.

