# Supplementary material for: Titration of RAS alters senescent state and influences tumour initiation
Source: Nature. 2024 Aug 7;633(8030):678–85. doi: 10.1038/s41586-024-07797-z (PMC11410659; doi:10.1038/s41586-024-07797-z)

nature portfolio

https://doi.org/10.1038/s41586-024-07797-z

Supplementary information

Titration of RAS alters senescent state and influences tumour initiation

In the format provided by the authors and unedited

Supplementary information Guide

Titration of RAS alters senescent state and influences tumour initiation

Adelyne S.L. Chan[1†], Haoran Zhu[1†], Masako Narita[1], Liam D. Cassidy[1], Andrew R.J. Young[1], Camino Bermejo-Rodriguez[2], Aleksandra T. Janowska[1], Hung-Chang Chen[1], Sarah Gough[1], Naoki Oshimori[3], Lars Zender[4,5,6,7], Sarah J. Aitken[1,8,9], Matthew Hoare[1,10,11], Masashi Narita[1,12*]

[1]Cancer Research UK Cambridge Institute, Li Ka Shing Centre, University of Cambridge, Cambridge, UK

[2]Department of Molecular and Clinical Cancer Medicine, University of Liverpool, L69 3GE, Liverpool, UK

[3]Department of Cell, Developmental & Cancer Biology, Knight Cancer Institute, Oregon Health and Science University, Portland, OR, USA

[4]Department of Medical Oncology and Pneumology, University Hospital Tuebingen, Tuebingen, Germany

[5]German Cancer Research Consortium (DKTK), Partner Site Tübingen, German Cancer Research Center (DKFZ), Heidelberg, Germany

[6]iFIT Cluster of Excellence EXC 2180 Image Guided and Functionally Instructed Tumor Therapies, University of Tuebingen, Tuebingen, Germany

[7] Tuebingen Center for Academic Drug Discovery & Development (TüCAD2)

[8]Medical Research Council Toxicology Unit, University of Cambridge, Cambridge, UK

[9]Department of Histopathology, Cambridge University Hospitals NHS Foundation Trust, Cambridge, UK

[10]Early Cancer Institute, Hutchison Research Centre, University of Cambridge, Cambridge, UK

[11]Department of Medicine, University of Cambridge, Cambridge, UK

[12]Tokyo Tech World Research Hub Initiative (WRHI), Institute of Innovative Research, Tokyo Institute of Technology, Yokohama, Japan

*Corresponding author. Email: Masashi.Narita@cruk.cam.ac.uk

† These authors contributed equally to this work.

Table of Contents:

1) Supplementary figures.pdf

- Supplementary Fig. 1. Gating and Sorting Strategy
  - Immune cell profiling in mouse liver
  - Predictive reporter system in vitro
  - Isolating mVenus-Expressing Hepatocytes for scRNA-seq
- Supplementary Fig. 2. Uncropped Western Blots
  - Original gel images for Fig.2d
  - Original gel images for ED Fig.3d

2) Source Data.xlsx

Individual raw data points and statistical analysis.

- Fig. 2
  - Figure 2e: SA-b-gal counts per subpopulation
  - Figure 2f: BrdU counts per subpopulation
  - Figure 2h: BrdU counts after S-phase enrichment by flow sorting
- Fig. 3
  - Figure 3b: % NRAS +ve area
  - Figure 3e: Cell number / g of liver for indicated immune cells subpopulations
  - Figure 3i: % of positive cells surrounded by immune cluster
- ED Fig. 3
  - Extended Data Figure 3f (top): SA-b-gal counts per subpopulation
  - Extended Data Figure 3f (bottom): BrdU counts per subpopulation
  - Extended Data Figure 3g: p-values for quantification of gamma-H2AX per cell
- ED Fig. 6
  - Extended Data Figure 6a: % gH2AX foci>1
  - Extended Data Figure 6d: % Foxp3 +ve area
- ED Fig. 7
  - Extended Data Figure 7b: % Dlk1 +ve area
- ED Fig. 8
  - Extended Data Figure 8b: % +ve area for indicated marker

3) Supplementary Table 1.xlsx

- Full names of DNA Damage-related gene sets from MsigDB (ED Fig. 1e)

4) Supplementary Table 2.xlsx

- Datasets & GSE IDs: Details for publicly available datasets, including download URLs, used for the meta-analysis in Extended Data Fig. 5a,b,e.

5) Supplementary Table 3.xlsx

- Cirrhosis patient demographic data in Extended Data Fig. 10

Supplementary Fig. 1. Gating and Sorting Strategy

1. Immune cell profiling in mouse liver

Representative flow cytometry plots detailing the gating strategy to identify 1) NK cells; 2) CD3+ CD4+; and 3) CD3+ CD8+ T cells.

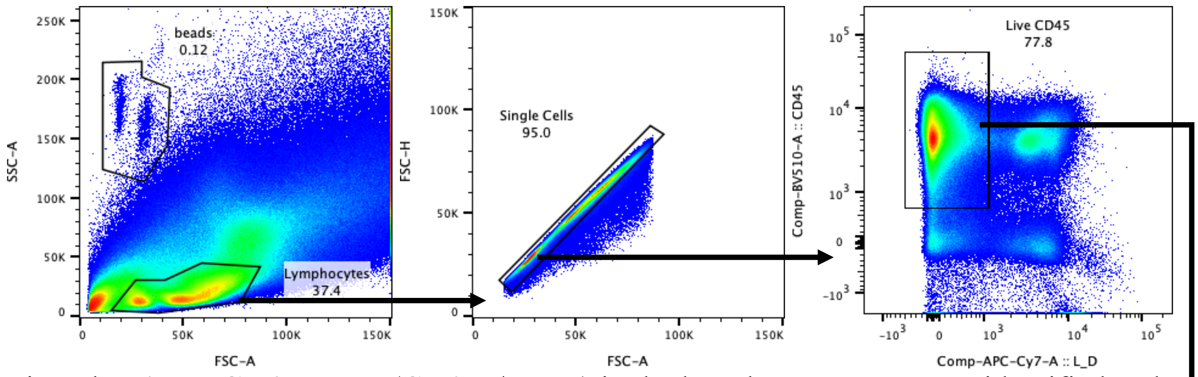

First, the **viable CD45+ cells** (CD45+/ L_D-) in the lymphocytes gate were identified and further sub-gated. L_D, Live_Dead (Fixable Viability Dye eFluor 780, eBioscience)

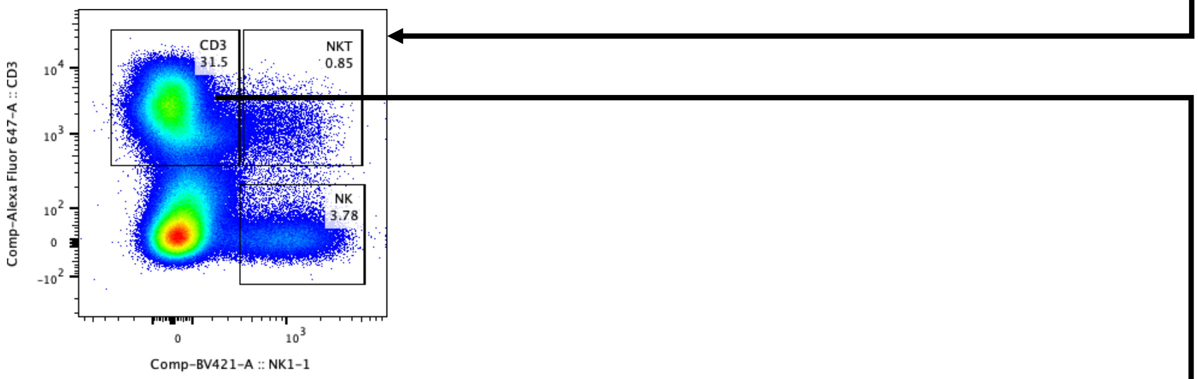

1) **NK cells**: The number of cellular events in CD3-/NK1.1+ lower right gate was used to determine the absolute number of NK cells. Identical gates were used for all samples analysed.

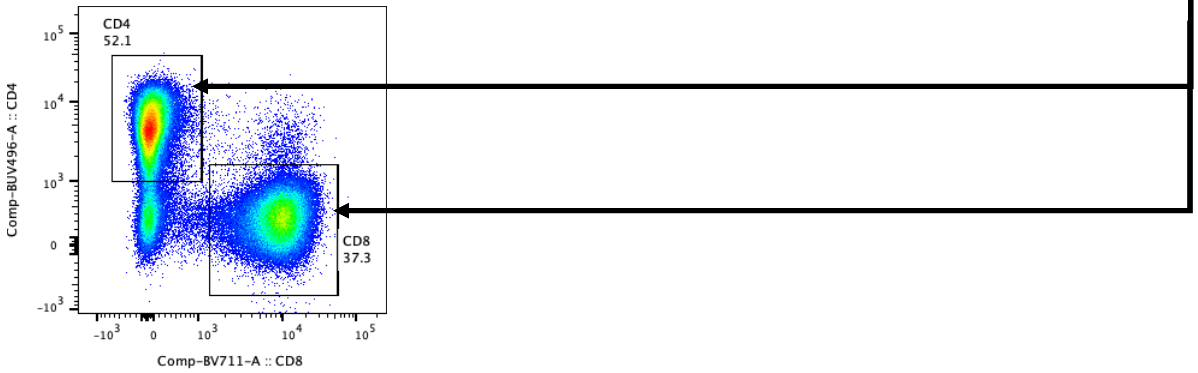

2) **CD4+ T cells:** The number of cellular events in the CD4+/CD8- upper left gate was used to determine the absolute number of CD3+CD4+ T cells.

3) **CD8+ T cells:** CD4-/CD8+ lower right gate was used to determine the absolute number of CD3+CD8+ T cells.

AccuCheck Counting Beads for absolute cell number assessment:

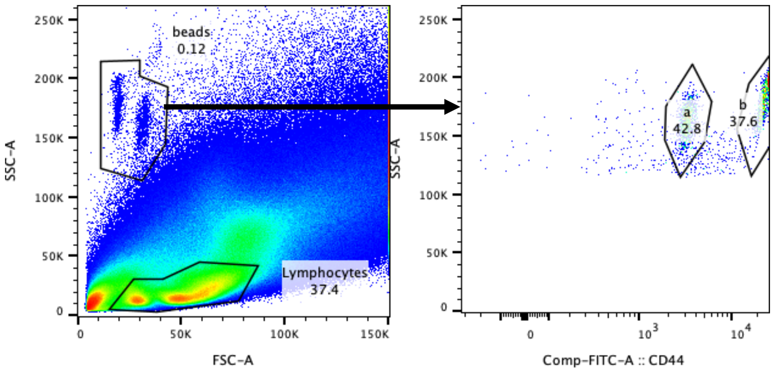

The number of events in gates for beads, a and b, were used to normalize absolute cell events processed from each sample. Identical gates were used for all samples analyzed.

AccuCheck Counting Beads for absolute cell number assessment:

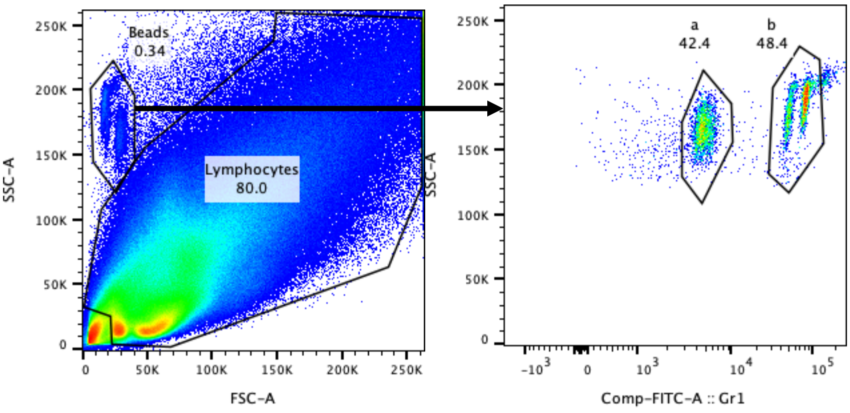

The number of events in gates for beads, a and b, were used to normalize absolute cell events processed from each sample. Identical gates were used for all samples analyzed.

2. Predictive reporter system in vitro

Quality Control (Representative gating strategy used for all samples)

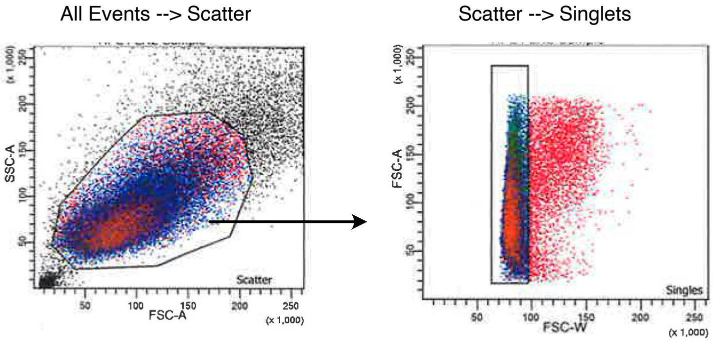

Sorting singlets into subpopulations by mVenus intensity was performed after the quality control gating steps shown above.

In RPE1 cells, we sorted parent populations expressing either the retroviral or the lentival construct into 3 and 4 subpopulations respectively (top panel). To further enrich for the highest expressors, we then employed a serial sorting approach of re-sorting the highest-expressing lentiviral subpopulation into a further 3 subpopulations (sort 2, bottom panel).

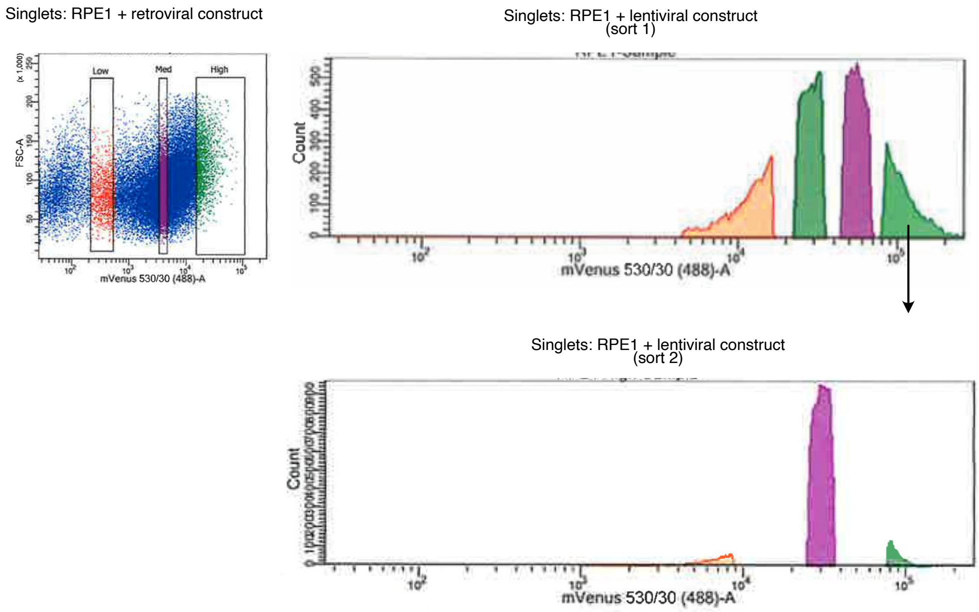

TIG3 subpopulations were generated from a single mixed parent. Briefly, cells expressing the retroviral or the lentiviral construct were generated separately (like with RPE1), then the populations were mixed at a 1:1 ratio just prior to sorting. This mixed parent population was then flow-sorted into 4 subpopulations.

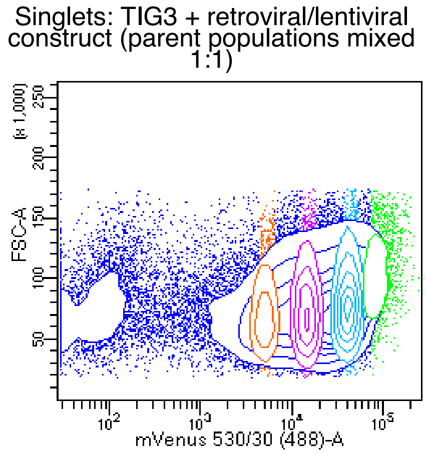

3. Isolating mVenus-Expressing Hepatocytes for scRNA-seq

After dissociation into single-cell suspension, isolated hepatocytes were labelled with Fixable Viability Dye eFluor 780 (eBioscience) and CD45 AlexaFluor 660 (eBioscience). On the flow sorter, cells were first gated according to the same quality control steps for scatter and singlets (the first two panels on the left).

Singlets were then gated to exclude dead cells (high expression of Live/Dead marker on 780/60 channel, panel 3) and mVenus positivity (530/30 channel, panel 4).

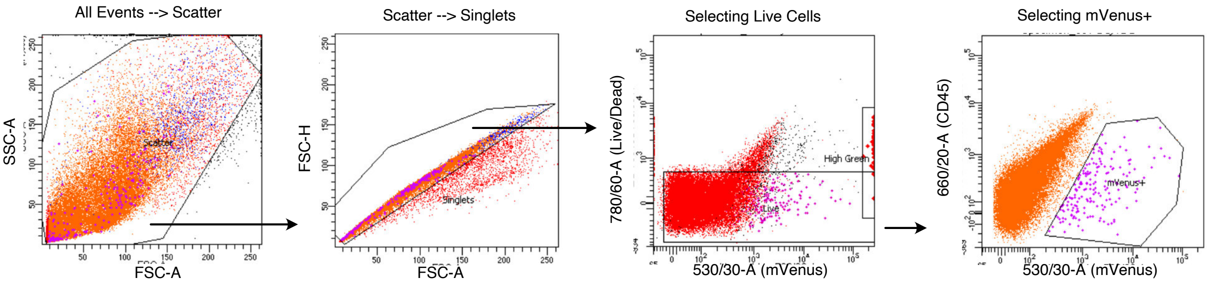

Supplementary Fig. 2. Uncropped Western Blots

Fig. 2d

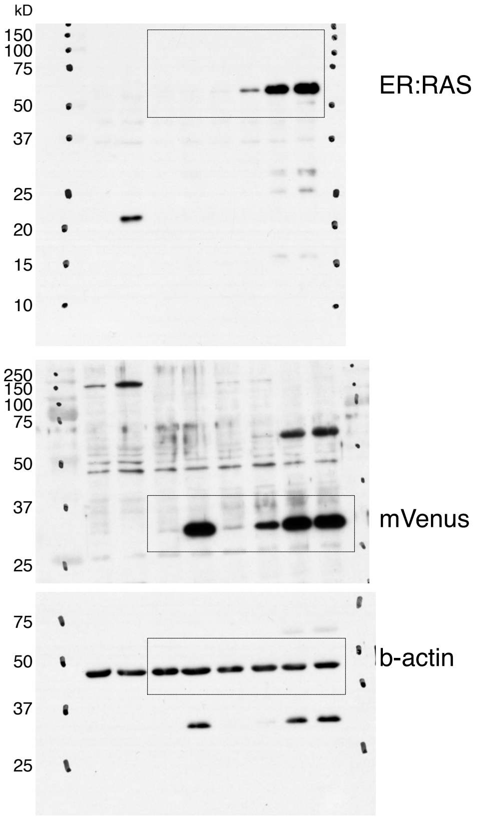

ED Fig. 3d

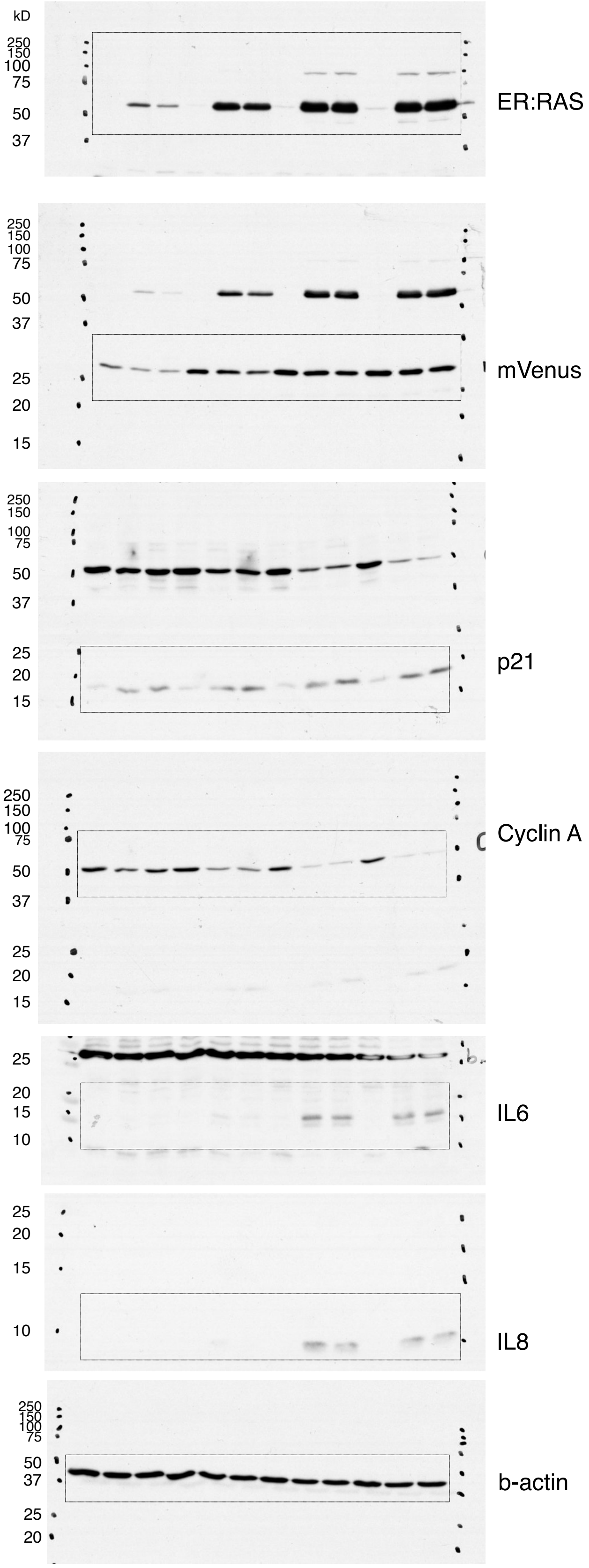

Supplement: Supplementary file 1 — This file contains Supplementary Fig. 1: Gating and sorting strategy; and Supplementary Fig. 2: Uncropped western blots for Fig. 2d and Extended Data Fig. 3d. [file 41586_2024_7797_MOESM1_ESM.pdf]
