# Supplementary material for: Titration of RAS alters senescent state and influences tumour initiation
Source: Nature. 2024 Aug 7;633(8030):678–85. doi: 10.1038/s41586-024-07797-z (PMC11410659; doi:10.1038/s41586-024-07797-z)
Supplement: Supplementary file 2 — Reporting Summary [file 41586_2024_7797_MOESM2_ESM.pdf]

nature portfolio

Corresponding author(s): Masashi Narita

Last updated by author(s): Jun 18, 2024

Reporting Summary

Nature Portfolio wishes to improve the reproducibility of the work that we publish. This form provides structure for consistency and transparency in reporting. For further information on Nature Portfolio policies, see our Editorial Policies and the Editorial Policy Checklist.

Statistics

For all statistical analyses, confirm that the following items are present in the figure legend, table legend, main text, or Methods section.

n/a	Confirmed	
☐	☒	The exact sample size (*n*) for each experimental group/condition, given as a discrete number and unit of measurement
☐	☒	A statement on whether measurements were taken from distinct samples or whether the same sample was measured repeatedly
☐	☒	The statistical test(s) used AND whether they are one- or two-sided<br>*Only common tests should be described solely by name; describe more complex techniques in the Methods section.*
☒	☐	A description of all covariates tested
☐	☒	A description of any assumptions or corrections, such as tests of normality and adjustment for multiple comparisons
☐	☒	A full description of the statistical parameters including central tendency (e.g. means) or other basic estimates (e.g. regression coefficient) AND variation (e.g. standard deviation) or associated estimates of uncertainty (e.g. confidence intervals)
☐	☒	For null hypothesis testing, the test statistic (e.g. *F*, *t*, *r*) with confidence intervals, effect sizes, degrees of freedom and *P* value noted<br>*Give P values as exact values whenever suitable.*
☒	☐	For Bayesian analysis, information on the choice of priors and Markov chain Monte Carlo settings
☒	☐	For hierarchical and complex designs, identification of the appropriate level for tests and full reporting of outcomes
☒	☐	Estimates of effect sizes (e.g. Cohen's *d*, Pearson's *r*), indicating how they were calculated

Our web collection on statistics for biologists contains articles on many of the points above.

Software and code

Policy information about availability of computer code

Data collection	IF images were collected using LAS X 3.7.5.24914 or LAS X 4.7.0 software (Leica).
Data analysis	Analysis and Visualisation of flow cytometry data: FlowJo (v10.9.0). RNAseq: FastQC (https://www.bioinformatics.babraham.ac.uk/projects/fastqc/) and Cutadapt (v1.10) for pre-processing, STAR aligner (v2.7.6a) and subread (v1.5.3) for alignment and feature counting, edgeR (v3.20.9) for differential analysis. scRNAseq: CellRanger (v3.1.0) for alignment, Seurat (v4.0.4), Monocle (v2.26.0 and v3.16.0) for analysis. General statistical analysis: GraphPad Prism 10.2.1, General data visualisation: ggplot2 (v3.4.2). Heatmaps: pheatmap (v1.0.12), Upset plot: UpsetR (v1.4.0). Survival analysis: survminer (v0.4.7). Geneset Enrichment Analysis: fgsea (v3.18).

For manuscripts utilizing custom algorithms or software that are central to the research but not yet described in published literature, software must be made available to editors and reviewers. We strongly encourage code deposition in a community repository (e.g. GitHub). See the Nature Portfolio guidelines for submitting code & software for further information.

Data

Policy information about availability of data

All manuscripts must include a data availability statement. This statement should provide the following information, where applicable:

- Accession codes, unique identifiers, or web links for publicly available datasets
- A description of any restrictions on data availability
- For clinical datasets or third party data, please ensure that the statement adheres to our policy

Liver OIS scRNAseq (Fig. 1b-g), RPE1 RNAseq (Fig. 2 and Extended Data Fig. 2), RPE1 scRNAseq (Extended Data Fig. 5 c-d) and liver tumorigenesis scRNAseq (Fig. 3) have been deposited at GEO (GSE222339, GSE222337, GSE249489 and GSE222338 respectively, under the Super Series GSE222951). Pancreatic timecourse scRNAseq data was downloaded from GSE141017. TCGA data was downloaded from the National Cancer Institute's GDC Portal (https://gdc.cancer.gov). Senescence-associated RNAseq data (Extended Data Fig. 3a) was downloaded from GEO: GSE74324, GSE61130, GSE72407, GSE72404, GSE127116, GSE45833, GSE85082, GSE45833, GSE63577, GSE53356. Squamous Cell Carcinoma tumor-initiating cell data (Extended Data Fig. 3b) was downloaded from GSE151783. The reference human (hg38) and mouse (mm10) genomes were downloaded from ensembl.org.

Research involving human participants, their data, or biological material

Policy information about studies with human participants or human data. See also policy information about sex, gender (identity/presentation), and sexual orientation and race, ethnicity and racism.

Reporting on sex and gender	Sex information was collected for human cirrhosis patients, with consent for sharing of anonymised individual-level data. No significant difference in phenotype was observed between sex in this cohort, therefore sex information was not considered for further analyses.
Reporting on race, ethnicity, or other socially relevant groupings	Ethnicity information was collected for human cirrhosis patient, with consent for sharing of anonymised individual-level data. However, as this patient cohort lacks the power to systematically study differences in phenotype by ethnicity, and this point is also not the focus on the current manuscript, ethnicity information was not considered in any of the analyses of the current study.
Population characteristics	Population characteristics of patients were collected and summarised in Supplementary Table 3.
Recruitment	Tissue sections were obtained from patients undergoing liver transplantation, with either alcohol-related liver disease, nonalcoholic fatty liver disease or hepatitis C-associated liver disease (indicated in Supplementary Table 3).
Ethics oversight	All biological samples were collected with informed consent from Addenbrooke's Hospital, Cambridge, UK, according to procedures approved by the Office for Research Ethics Committees Northern Ireland (ORECNI) (20/NI/0109). All participants consented to publication of research results. This information is indicated in the Methods section of the manuscript.

Note that full information on the approval of the study protocol must also be provided in the manuscript.

Field-specific reporting

Please select the one below that is the best fit for your research. If you are not sure, read the appropriate sections before making your selection.

☒ Life sciences ☐ Behavioural & social sciences ☐ Ecological, evolutionary & environmental sciences

For a reference copy of the document with all sections, see nature.com/documents/nr-reporting-summary-flat.pdf

Life sciences study design

All studies must disclose on these points even when the disclosure is negative.

Sample size	For SAbgal and BrdU percent positivity, sample size was based on our previous study (PMID16901784). At least 200 cells were counted per condition from >3 biological replicates. Western blots and flow cytometry are reproduced in at least 3 independent experiments. Quantification for IF experiments was performed in at least 2 independent experiments. For HDTVi studies and associated experiments e.g. tumour incidence, IHC, sample size was based on our previous study (PMID27525720). For IHC percent positivity, whole liver positivity was measured from >3 biological replicates per condition. Intensity was measured from at least 200 cells per liver, from >3 biological replicates. For tumour incidence experiments, data are shown from >9 mice per condition. Human liver cirrhosis data is based on 28 patients.
Data exclusions	No data was excluded from the analysis
Replication	Liver OIS scRNAseq: 2 mice (G12V) and 1 mouse (D38A) respectively. RPE1 RNAseq: 5 biological replicates per condition. RPE1 scRNAseq: 1 monocultured biological replicate per subpopulation, plus a pooled second replicate (and uninduced control). Liver tumorigenesis scRNAseq: 2 mice per timepoint. Western blots, immunofluorescence, IHC and flow cytometry are reproduced in at least 3 independent experiments. All attempts at replication were successful.
Randomization	Plate layout randomisation was applied for RNAseq library preparation. For all scRNAseq experiments, cells from the different conditions were pooled prior to library prep then processed as a single sample, therefore randomisation is not possible nor necessary. For in vivo experiments, mice were randomised into groups for HDTV with the different constructs in each experiment. For in vitro experiments, the nature of the

experimental setup is that dose-dependency is established in pre-defined subpopulations prior to RAS induction, therefore randomisation is not possible. Cells were randomised into "uninduced" and "induced" plates on the day of induction.

Blinding	Tumours were graded by a board-certified pathologist blinded to experimental design. Mouse liver tumour palpation for Kaplan-Meier analysis was performed weekly by an animal technician blinded to experimental condition. DLK1/NOTCH1 status of human samples was assessed with no knowledge of patient clinical information. No blinding was applied to other experiments, where data analysis are based on objectively measurable data i.e. quantifiable measurements.

Reporting for specific materials, systems and methods

We require information from authors about some types of materials, experimental systems and methods used in many studies. Here, indicate whether each material, system or method listed is relevant to your study. If you are not sure if a list item applies to your research, read the appropriate section before selecting a response.

Materials & experimental systems

n/a	Involved in the study
☐	☒ Antibodies
☐	☒ Eukaryotic cell lines
☒	☐ Palaeontology and archaeology
☐	☒ Animals and other organisms
☐	☒ Clinical data
☒	☐ Dual use research of concern
☒	☐ Plants

Methods

n/a	Involved in the study
☒	☐ ChIP-seq
☐	☒ Flow cytometry
☒	☐ MRI-based neuroimaging

Antibodies

Antibodies used	anti-GFP (Abcam ab13970), anti-Ras (Abcam ab52939, EP1125Y), anti-p-Erk1/2 (Cell Signaling Technology #9101), anti-CK8 (DSHB MABT329, TROMA-1), anti-CK19 (DSHB MABT913, TROMA-III), anti-mouse Nestin (Chemicon MAB353, rat-401), anti-human Nestin (Chemicon MAB5326, 10C2), anti-Afp (Santa Cruz sc-8399, C3), anti-mouse Dlk1 (R&D Systems #FAB8634T, 1168B), anti-human DLK1 (R&D Systems MAB1144, 211309), anti-Notch1 (Cell Signaling Technology #3608, D1E11), anti-TGF beta (Cell Signaling Technology #3709, 56E4), anti-mouse CD4 (Abcam ab183685, EPR19514), anti-mouse CD8α (Cell Signaling Technology #98941, D4W2Z), anti-mouse F4/80 (Serotec MCA497, CLA3-1), anti-mouse FoxP3 (eBioscience 14-5773, FJK-16s), anti-human CD4 (Dako M7310, 4B12), anti-human CD8 (Thermo Fisher Scientific RM-9116-S, SP16), anti-human CD68 (Novocastra NCL-L-CD68, 514H12), anti-BrdU (BD Biosciences Cat # 555627, 3D4), anti-phospho-Histone H2A.X (Ser139) (Merck Cat # 05-636, JBW301), anti-b-actin (Sigma Cat # A5441, AC15), anti-HRAS (Santa Cruz Biotechnology Cat # sc29, F235), anti-GFP (Clontech Cat # 632377), anti-IL6 (R&D Biosystems Cat # MAB2061, Clone#1936), anti-IL8 (R&D Biosystems Cat # MAB208, Clone#6217), anti-Cyclin A (Sigma Cat # c4710, CY-A1), anti-p21 (Santa Cruz Cat # sc-6246, F5)
Validation	Anti-b-actin (A5441, Sigma) Used for: WB. Species Against: Human. This antibody was validated by the company and used in our previous studies (PMID27525720, PMID29743479).<br>Anti-HRAS (sc29, Santa Cruz Biotechnology) Used for: WB. Species Against: Human. This antibody was validated by the company and used in our previous study (doi:10.1038/s43587-021-00147-y).<br>Anti-GFP (632377, Clontech) Used for: WB. Species Against: Human. This antibody was validated by the company and used in our previous study (PMID16901784).<br>Anti-IL6 (MAB2061, R&D Biosystems) Used for: WB. Species Against: Human. This antibody was validated by the company and used in our previous study (PMID33730589).<br>Anti-IL8 (MAB208, R&D Biosystems) Used for: WB, IF. Species Against: Human. This antibody was validated by the company and used in our previous study (PMID33730589).<br>Anti-Cyclin A (c4710, Sigma) Used for: WB. Species Against: Human. This antibody was validated by the company.<br>Anti-p21 (sc-6246, Santa Cruz) Used for: WB. Species Against: Human. This antibody was validated by the company.<br>Anti-phospho-Histone H2A.X (05-636, Merck) Used for: IF. Species Against: Human, Mouse. This antibody was validated by the company.<br>Anti-BrdU (555627, BD Biosciences) Used for: IF. Species Against: Human. This antibody was validated by the company and used in our previous study (PMID33730589).<br>Anti-Nras (ab52939. Abcam) Used for: IHC. Species Against: Mouse. This antibody was validated by the company and used in our previous study (PMID27525720).<br>Anti-GFP (ab13970, Abcam) Used for: IHC. Species Against: Mouse. This antibody was validated by the company.<br>Anti-Notch1 (3608, Cell Signalling Technology) Used for: IHC. Species Against: Mouse. This antibody was validated by the company and used in our previous study (PMID27525720).<br>Anti-Nestin (MAB353, Chemicon) Used for: IHC. Species Against: Mouse. This antibody was validated by the company.<br>Anti-Nestin (MAB5326, Chemicon) Used for: IHC. Species Against: Mouse. This antibody was validated by the company.<br>Anti-Dlk1 (FAB8634T, R&D Systems) Used for: IHC. Species Against: Mouse. This antibody was validated by the company.<br>Anti-DLK1 (MAB1144, R&D Systems) Used for: IHC. Species Against: Mouse. This antibody was validated by the company.<br>Anti-Afp (sc8399, Santa Cruz Biotechnology) Used for: IHC. Species Against: Mouse. This antibody was validated by the company.<br>Anti-CK19 (TROMA-III, DSHB) Used for: IHC. Species Against: Mouse. This antibody was validated by the company.<br>Anti-CK8 (TROMA-I, DSHB) Used for: IHC. Species Against: Mouse. This antibody was validated by the company.<br>Anti-pErk1/2 (9101, Cell Signalling Technology) Used for: IHC. Species Against: Mouse. This antibody was validated by the company.<br>Anti-TGF beta (3709, Cell Signaling Technology) Used for: IHC. Species Against: Mouse. This antibody was validated by the company.<br>Anti-mouse CD4 (ab183685, abcam) Used for: Flow. Species Against: Mouse. This antibody was validated by the company.

Anti-mouse CD8a (98941, Cell Signaling Technology) Used for: Flow. Species Against: Mouse. This antibody was validated by the company.
Anti-mouse F480 (MCA497, Serotec) Used for: Flow. Species Against: Mouse. This antibody was validated by the company.
Anti-mouse FoxP3 (14-5773, eBioscience) Used for: Flow. Species Against: Mouse. This antibody was validated by the company.
Anti-human CD4 (M7310, Dako) Used for: Flow. Species Against: Human. This antibody was validated by the company.
Anti-human CD8 (RM-9116-S, Thermo Fisher Scientific) Used for: Flow. Species Against: Human. This antibody was validated by the company.
Anti-human CD68 (NCL-L-CD68, Novocastra) Used for: Flow. Species Against: Human. This antibody was validated by the company.
Anti-TGFbeta (3709, Cell Signaling Technology) Used for: IHC. Species Against: Mouse. This antibody was validated by the company.

Eukaryotic cell lines

Policy information about cell lines and Sex and Gender in Research

Cell line source(s)	RPE1-hTert (ATCC), TIG3 (JCRB)
Authentication	Cells were obtained directly from the respective source cell banks. No authentication was performed by the authors of this manuscript.
Mycoplasma contamination	Cells were regularly tested for Mycoplasma contamination and always found to be negative.
Commonly misidentified lines (See ICLAC register)	No commonly misidentified lines were used in this study.

Animals and other research organisms

Policy information about studies involving animals; ARRIVE guidelines recommended for reporting animal research, and Sex and Gender in Research

Laboratory animals	C57BL/6 and CB17/Icr-Prkdcscid/IcrIcoCrl mice were used in this study. HDTV injections were carried out on mice between 6 and 8 weeks of age.
Wild animals	No wild animals were used in this study.
Reporting on sex	Only female mice were used in all experiments apart from in the sex comparison long-term cohort, where one cohort of male mice was used.
Field-collected samples	No field-collected samples.
Ethics oversight	The CRUK CI Animal Welfare and Ethics Review Board (AWERB; Institutional Animal Care and Use Committee) approved all animal experiments performed in this study. All animal work was conducted in accordance with UK law.

Note that full information on the approval of the study protocol must also be provided in the manuscript.

Clinical data

Policy information about clinical studies
All manuscripts should comply with the ICMJE guidelines for publication of clinical research and a completed CONSORT checklist must be included with all submissions.

Clinical trial registration	No active clinical trials were performed in this study.
Study protocol	No active clinical trials were performed in this study.
Data collection	Tissue sections were obtained from patients undergoing liver transplantation, with either alcohol-related liver disease, nonalcoholic fatty liver disease or hepatitis C-related liver disease (indicated in Supplementary Table 3).
Outcomes	No active clinical trials were performed in this study.

Plants

Seed stocks

Report on the source of all seed stocks or other plant material used. If applicable, state the seed stock centre and catalogue number. If plant specimens were collected from the field, describe the collection location, date and sampling procedures.

Novel plant genotypes

Describe the methods by which all novel plant genotypes were produced. This includes those generated by transgenic approaches, gene editing, chemical/radiation-based mutagenesis and hybridization. For transgenic lines, describe the transformation method, the number of independent lines analyzed and the generation upon which experiments were performed. For gene-edited lines, describe the editor used, the endogenous sequence targeted for editing, the targeting guide RNA sequence (if applicable) and how the editor was applied.

Authentication

Describe any authentication procedures for each seed stock used or novel genotype generated. Describe any experiments used to assess the effect of a mutation and, where applicable, how potential secondary effects (e.g. second site T-DNA insertions, mosiacism, off-target gene editing) were examined.

Flow Cytometry

Plots

Confirm that:

☒ The axis labels state the marker and fluorochrome used (e.g. CD4-FITC).

☒ The axis scales are clearly visible. Include numbers along axes only for bottom left plot of group (a 'group' is an analysis of identical markers).

☒ All plots are contour plots with outliers or pseudocolor plots.

☒ A numerical value for number of cells or percentage (with statistics) is provided.

Methodology

Sample preparation

RPE1-hTert and TIG3 cells grown in culture were trypsinised into suspension and immediately run on the flow sorter. Hepatocytes and immune cells were isolated from the liver using a collagenase-based dissociation protocol and immediately run on the flow sorter.

Instrument

Analysis of fluorochrome intensity was performed on a MacsQuantVYB (Miltenyi Biotech) for RPE1 and TIG3 experiments, and on a LSRFortessa Cell Analyzer or BD FacsSymphony (BD) for the mouse immune profiling experiments. Cell sorting was performed using a FACS Aria sorter (Becton Dickinson).

Software

Analysis was performed using FlowJo (v10.9.0, Becton Dickinson)

Cell population abundance

Abundance of cell populations post-sort ranged from 2% to 50% depending on application. Purity of fractions was determined by returning cells to culture and then re-running flow cytometry analysis 7-30 days post-analysis to demonstrate separation of subpopulations established by the flow sorting experiment.

Gating strategy

Debris was gated out using FSC-A/SSC-A gate, then singlets were selected for using FSC-H/FSC-A gate. For in vivo preparations, a live/dead dye was added for gating out unviable cells.

☒ Tick this box to confirm that a figure exemplifying the gating strategy is provided in the Supplementary Information.
